# The predictive capability of several anthropometric indices for identifying the risk of metabolic syndrome and its components among industrial workers

**DOI:** 10.1038/s41598-024-66262-z

**Published:** 2024-07-03

**Authors:** Ekaterina D. Konstantinova, Tatiana A. Maslakova, Svetlana Yu. Ogorodnikova

**Affiliations:** Institute of Industrial Ecology of Ural Branch of the Russian Academy of Sciences, S. Kovalevskaya Str., 20, Ekaterinburg, 620990 Russian Federation

**Keywords:** Metabolic syndrome, Anthropometric index, ROC analyses, Logistic regression, Body mass index, Occupational health, Prognostic markers, Statistics

## Abstract

Metabolic syndrome (MetS) is closely associated with adverse cardiometabolic outcomes. The objective of this study was to identify practical methods that could enable the effective identification of MetS based on anthropometric indices. The basis of our study involved retrospective database obtained from routine medical prophylactic examinations. This was a cross-sectional study on the health status of male workers employed in hazardous working conditions at industrial enterprises in the Ural region conducted in 2019. A total of 347 male workers employed under hazardous working conditions were investigated. The presence of MetS was established by a healthcare professional in accordance with the guidelines of the International Diabetes Federation (IDF). Simple linear regression was used to evaluate the associations between anthropometric indices and MetS incidence. Logistic regression was used to determine the odds ratios of MetS in relation to increases in anthropometric indices. ROC curves were calculated to compare the ability of each anthropometric index to predict MetS and to determine the diagnostic thresholds of the indicators considered. According to the IDF criteria, 36.3% of the workers had MetS. A direct relationship was found between the individual components of MetS and the anthropometric indices studied. The highest OR was shown by the Body Roundness Index (BRI) of 2.235 (95% CI 1.796–2.781). For different age quartiles, the optimal cut-off values for predicting MetS were as follows: BRI, 4.1–4.4 r.u.; body shape index (ABSI), 0.080–0.083 m^11/6^ kg^−2/3^; and lipid accumulation product (LAP), 49.7–70.5 cm mmol/l. The most significant associations with MetS were observed where the values were greater than these cut-off points (Se = 97.4%). The results of this study demonstrated the rapid use of new anthropometric indicators, which have shown good predictive ability and are quite easy to use.

## Introduction

Several cardiometabolic risk factors are known to be involved in metabolic syndrome, and MetS is an important predictor of all-cause mortality^1^. The risk factors present in metabolic syndrome patients are mentioned below. According to the World Health Organization (WHO), approximately 25–30% of the adult population in different countries worldwide currently suffer from MetS. In addition, it is predicted that this figure will continue to grow in the next 10 years^2^. According to a number of experts, MetS is a symptom of broader changes in society, such as urbanization and lower physical activity. These factors provoke the development of obesity and other MetS components^3^.

Recent studies have provided strong evidence that MetS is a serious worldwide threat and poses a difficult problem for health workers. The rapid increase in the prevalence of MetS foreshadows a corresponding frightening increase in its harmful consequences, namely, type 2 diabetes mellitus and cardiovascular diseases, which cause significant economic damage at the population level worldwide^4^. A significant part of the impact of obesity on the economy as a whole is associated with a decrease in labor productivity and a reduction in human capital. In the case of employment, persons with chronic diseases will be absent from work for 1.5% more days during the remainder of their working life^5^.

It is worth emphasizing that the majority of individuals with MetS are in the active working age group and represent a productive and significant part of society. According to the WHO, the world is facing a new pandemic in the twenty-first century, spreading across industrialized countries. This could also constitute a demographic catastrophe for developing countries^6^.

The underlying factors of MetS are insulin resistance and central obesity^7^. Anthropometry is the simplest, most economical and widespread method of obtaining information that allows one to judge the characteristics of human physical development and nutrition. By comparing specific values of anthropometric indicators with established standards, a specialist can make a primary conclusion about the presence of certain deviations and decide on the need for in-depth research.

Body mass index (BMI) provides reliable information on overweight status but does not reflect the nature of fat tissue distribution, the fat-to-muscle weight ratio, etc.^8–10^. Waist circumference reflects visceral or abdominal obesity better than BMI^11^. Moreover, waist circumference (WC) classified the majority of obese people as suffering from abdominal obesity. In addition, WC and hip circumference are strongly correlated with BMI^12^.

Moreover, the type of adipose tissue distribution is closely related to the incidence of arterial hypertension, diabetes mellitus, atherosclerosis, and MetS^8^.

The need for a more accurate representation of adipose tissue distributions led to the development of new informative indicators: BRI, ABSI, and LAP.

The BRI was proposed in 2013^13^. Some authors believe that the BRI has the ability to predict the incidence of MetS in the general population, among obese and overweight individuals, and among postmenopausal women^14,15^.

The ABSI was suggested in 2012^16^. Because it is capable of revealing the relationships between WC and height and weight, the ABSI is considered to be a quantitative measure of body shape. Whereas BMI is related to body size, ABSI may be regarded as an indicator of “body shape roundness”^17^. Above-average ABSI values were found to be associated with a significantly greater risk of death. Previous studies have also shown that a high mortality risk is more strongly correlated with the ABSI than with BMI or WC^16^.

The term LAP was first mentioned by Kahn^18^, who considered this index a marker of excessive lipid accumulation in adults. The calculation of LAP is based on two simple measures: WC and fasting plasma triglyceride levels. LAP is used as a marker of serious conditions, such as MetS, insulin resistance, diabetes mellitus, and nonalcoholic fatty liver disease, as well as the risk of stroke, hypertension, renal dysfunction, and cardiovascular pathologies^19–25^.

There is no doubt that occupational environments can have a significant impact on the lifestyle and health of workers. Several studies have shown a relationship between environmental factors at work and the occurrence of metabolic disorders^26,27^. Therefore, Loukzadeh et al. reported a significant association between a mean decrease in forced expiratory volume in 1 s in 5 years and MetS in dust-exposed workers^26^. In a recent study, Kim and colleagues showed that occupational noise contributed significantly to the onset of MetS and to changes in its components^27^.

Night shift work is also associated with metabolic risk factors^28^. Brum et al. showed that night work was a risk factor for abdominal obesity, that social jet lag was greater in night shift workers, and that it was associated with the presence of obesity^29^.

The prevalence of both obesity and MetS in the Russian Federation has been steadily increasing, which is motivating researchers to look for noninvasive and cost-effective methods to identify individuals at risk^30^. This is especially true in relation to industrial workers employed under hazardous working conditions. Not all corporate medical units can use expensive invasive procedures for diagnosis. Therefore, new effective and inexpensive indicators are needed to assess central obesity. The BRI, ABSI, and LAP are indices that are specially designed to meet these needs. Despite the obvious advantages of their use in identifying the risk of MetS, they have not yet become widespread in Russia.

We have examined the predictive power of BMI, the BRI, the ABSI, and LAP for identifying individuals with MetS and its components among industrial workers working in hazardous conditions. Early identification of at-risk individuals facilitates the development of risk factor handling programs and prevention of MetS onset and progression.

## Methods

### Study population

The study protocol was approved by the institutional supervision board (Protocol No. 3 from 05.06.2023). This research does not contradict medical ethical principles, particularly the provisions of the Helsinki Declaration, according to the wording of 2013. The research methodology and the informed consent of the participants were in compliance with the rules of evidence-based medicine, ethical standards and federal law requirements "On the protection of public health (1993)," and "On personal data" (2006). The survey and examination of participants were carried out on a voluntary basis after familiarizing them with the tasks and procedures of the study; consent to participate in the study was confirmed by the participants’ personal signatures. All personal data from the electronic databases were downloaded.

The inclusion criteria for individuals were as follows: provided written informed consent for participation in a research study; male; worked at the company under study in hazardous working conditions of the third class (harmful) 1–4°, depending on the equipment used; the technological lubricants used; and the duties performed on the team, causing different distances from the sources of generation of harmful production factors.

The exclusion criteria for patients were as follows: refusal to participate in the study and lack of routine medical check-up data.

According to the order of the Ministry of Labor of Russia dated July 18, 2019 N 512n^31^, the employer is not entitled to use the labor of women in jobs with harmful and dangerous working conditions or in underground work, with the exception of nonphysical work, work on sanitary and domestic services, training and internships. Therefore, this study was performed involving an exclusive population of individuals of only one sex (male). This study uses data from male workers employed in hazardous working conditions at industrial enterprises in the Ural region.

The leading factors in the working conditions of the production under study were an unfavourable microclimate (cooling and heating), noise, general and local vibration, electromagnetic fields, aerosols of predominantly fibrogenic action and harmful substances in the air of the working area (including copper, lead, and their compounds), heaviness, and tension in the labor process.

The clinical part of the study was carried out at the Federal Medical Research Center. The data were collected during 2019. A total of 347 workers aged 27–63 years were examined (mean age and standard deviation 46.5 ± 8.3 years).

### Data collection

The data were collected as part of a routine medical check-up. Information about age, length of employment in hazardous conditions, and presence of pathologies was collected from the case histories of the participants (current and past diseases, including diabetes mellitus and arterial hypertension). For all subjects, we measured height, weight, waist circumference, and hip circumference. Additionally, blood samples were taken.

WC was measured with an inelastic measuring tape. Participants were positioned standing, with their arms extended along the body; the tape was positioned between the upper edge of the iliac crest and the last rib. Three nonconsecutive measurements were taken; the first was discarded, and the average of the last two was considered in the final measurement.

Height and weight were measured with the participants in light clothing and without shoes. Blood pressure (BP) was measured three times in the sitting position after 5 min of rest, and the values of the second and third measurements were averaged and recorded. Blood samples were obtained by venipuncture after an 8-h fast. Serum triglyceride (TG) levels, fasting blood glucose levels, and high-density lipoprotein (HDL) levels were measured. Serum triglycerides and HDL were measured in a subgroup of 169 workers. Therefore, the LAP index was calculated for 169 workers.

The hemodynamic data collected included systolic blood pressure and diastolic blood pressure. Workers were instructed not to consume food, alcoholic beverages, or coffee 30 min before BP measurement; not to smoke 30 min before data collection; not to practice strenuous physical exercise in the previous 60 min; and to empty their bladder before performing the test. Participants were seated with their legs uncrossed and feet flat on the floor, with their backs resting on the chair, and relaxed. The right arm was positioned at the heart level and was supported, with the palm facing upwards, without clothing.

Anthropometric and arterial BP measurements and laboratory investigations were performed by trained medical personnel according to standardized protocols.

The variable by which the study sample was divided into groups was age. The data on professional activity included information about the profession and experience under harmful working conditions. Educational level data were collected using a questionnaire. All the workers studied had secondary or vocational education. Persons with a high education level were absent from the study sample. The lifestyle-related variables were height, weight, BMI, BRI, ABSI, and smoking. The medical history of each worker assumed an anamnesis of life, including information on smoking, smoking experience, and daily number of cigarettes. A person was considered to be a smoker according to the criteria used in the American Behavioral Risk Factor Surveillance System^32^. Therefore, in the present study, smokers were defined as people who smoked more than 100 cigarettes during their life and smoked daily or occasionally at the time of the study.

### Anthropometric indices

The anthropometric indices used in this analysis were BMI, BRI, ABSI, and LAP. The indices under study were calculated using the following formulas^13,16,18^:

1 $$ {\text{BMI = }}\frac{{{\text{weight}}}}{{{\text{height}}^{{2}} }} $$

2 $$ {\text{BRI = 364}}{.2} - {365}{\text{.5}}\sqrt {{1} - \frac{{{\text{WC}}^{{2}} }}{{{\uppi }^{{2}} \cdot {\text{height}}^{{2}} }}} $$

3 $$ {\text{ABSI = }}\frac{{{\text{WC}}}}{{{\text{BMI}}^{{\frac{{2}}{{3}}}} \cdot {\text{height}}^{{\frac{{1}}{{2}}}} }} $$

4 $$ {\text{LAP = (WC-65)}} \cdot {\text{Triglycerides}} $$

### Metabolic syndrome

The MetS components were determined according to the current IDF criteria^33^. These criteria specify the presence of central (abdominal) obesity and two or more additional clinical features. Abdominal obesity was defined as a WC ≥ 94 cm (males) or 80 cm (females).

The additional clinical signs of MetS were as follows:

- Elevated triglycerides ≥ 1.7 mmol/l;
- A HDL concentration for males < 1.03 mmol/l;
- BP ≥ 130/85 mmHg or had been diagnosed with arterial hypertension;
- Participants had a fasting blood glucose level ≥ 5.6 mmol/l or had been diagnosed with diabetes mellitus.

These determinants include a combination of categorical and borderline risk factors that can be easily measured in clinical practice.

### Statistical analysis

To compare the groups of workers with and without MetS, descriptive statistics were used (mean values and standard deviation, ${{\bar{\text{X}}}}$± SD for normally distributed indicators; medians and their percentiles, Me (P25-P75), for normally distributed indicators). Student's, Mann‒Whitney, and Kruskal‒Wallis tests were used to determine the statistical significance of differences in means and medians.

A heatmap approach helped visualize the clinical data of the workers. Simple linear regression was performed to evaluate associations between the anthropometric indices and MetS incidence. Logistic regression was used to calculate the MetS OR in relation to the variation in the anthropometric parameters. The ABSI was scaled up by a factor of 1000 because of its small range.

Statistical data processing was performed using Statistica for Windows v.10 (StatSoft, USA) and SPSS 29 (SPSS, Chicago, Illinois, USA) statistical software packages. The statistical significance of the differences was estimated at the significance levels α = 0.05 and α = 0.10.

### Limitations of this study

The present study has several limitations. First, the present study was a cross-sectional study, and we were unable to detect causal associations between anthropometric indices and MetS. Prospective studies are recommended to confirm the associations of anthropometric indices with MetS in the future. In addition, the current studies are limited to two diagnostic groups (nondisease and disease). Moreover, the present study focused on industrial workers, and the abilities of anthropometric obesity indices to predict MetS among populations with different working conditions. Meanwhile, the findings of the present study are only applicable to workers employed in hazardous working conditions in Russia, and the proposed optimal cut-off values might not be applicable to other overseas workers due to racial differences. Considering differences in body composition between ethnicities, more studies are needed to determine specific optimal cut-off values for other industrial workers. Russia is a multi-ethnic country. Notable differences in body composition between ethnicities could potentially confound the associations between anthropometric indices and MetS. Therefore, future studies are needed to determine cut-offs based on ethnicity. We were also unable to take into account a short-term factor in the analysis, namely, dietary energy intake.

## Results

The study sample included 347 male workers aged 27–63 years (mean age 46.5 ± 8.3 years) employed in hazardous working conditions. In the subject group, there were 45.2% of overweight individuals (BMI = 25.0–29.9 kg/m^2^), and 27.7% of individuals with obesity (BMI ≥ 30.0 kg/m^2^). Of these individuals, 36.3% had MetS according to the IDF criteria. The sample was divided into age quartiles. The first quartile (Q_1_) included 27- to 40-year-old subjects (mean age 35.3 ± 3.3 years); Q_2_ = 41- to 46-year-olds (mean age 43.4 ± 1.8 years); Q_3_ = 47- to 52-year-olds (mean age 49.6 ± 1.8 years); and Q_4_ = 53- to 63-year-olds (mean age 56.6 ± 2.8 years). The quartiles were proportionate in terms of the number of workers enrolled, which allowed reasonable conclusions to be drawn from group comparisons.

The main characteristics of the study sample by age group are presented in Table 1.

**Table 1:** Behavioural, anthropometric and clinical characteristics of workers by age group.

Parameter	Age, years				*p* value^a^
	Q_1_ 27–40 n = 84 (24.7%)	Q_2_ 41–46 n = 81 (23.8%)	Q_3_ 47–52 n = 87 (25.6%)	Q_4_ 53–63 n = 88 (25.9%)	
n (%)					
Metabolic syndrome	15 (17.9)	29 (35.8)	39 (42.9)	43 (47.3)	< 0.001
Abdominal obesity	43 (51.2)	53 (65.4)	70 (76.9)	67 (73.6)	0.002
Smoking (current)	56 (66.7)	50 (62.5)	66 (74.2)	39 (44.3)	> 0.05
${\bar{\text{X}}}$± SD					
BMI, kg/м^2^	26.4 ± 3.0	28.1 ± 3.4	28.9 ± 4.5	27.8 ± 3.8	0.012
WC, cm	93.5 ± 8.8	98.8 ± 10.0	102.2 ± 11.2	101.3 ± 10.7	< 0.001
Me (P25-P75)					
Glucose, mmol/l	5.5 (5.0–5.8)	5.6 (5.2–5.9)	5.9 (5.4–6.2)	5.7 (5.5–6.2)	0.003
Triglycerides, mmol/l	1.2 (1.0–1.9)	1.5 (1.0–2.2)	1.6 (1.1–2.2)	1.7 (1.3–2.5)	0.081
HDL, mmol/l	1.3 (1.1–1.4)	1.1 (1.0–1.4)	1.2 (1.0–1.4)	1.2 (1.0–1.4)	0.624
BRI, rel. units	4.0 (3.2–4.7)	4.4 (3.7–5.4)	5.0 (4.0–5.8)	5.1 (4.1–5.9)	< 0.001
ABSI, m^11/6^ kg^−2/3^	0.079 (0.077–0.082)	0.080 (0.078–0.083)	0.082 (0.080–0.084)	0.085(0.081–0.087)	< 0.001
LAP, cm∙mmol/l	40.3 (23.1–54.6)	50.5 (35.8–91.1)	65.5 (39.6–83.2)	65.6 (34.6–93.5)	< 0.001

^a^by the nonparametric Kruskal‒Wallis test was used to compare more than two groups.

The incidence of MetS increased monotonically from the first quartile to the fourth quartile (17.9% vs. 47.3%, *p* < 0.001), as did the medians of the BRI, ABSI and LAP indices (*p* < 0.001). The same trend was found for the length of employment in hazardous working conditions, as expected, as this indicator is strongly correlated with age. Smoking was more frequent in the third group than in the other quartiles (74.2% compared to 44.3% and 62.5%, respectively; *p* < 0.001). The subjects in the third quartile had the highest WC, BMI, and median blood glucose values. Furthermore, the third group had a greater percentage of individuals with abdominal obesity (WC > 94 cm) (76.9% compared to 51.2%, 65.4% and 73.6%, *p* < 0.05). There were no significant differences between the groups in terms of the median levels of triglycerides or HDL in the blood.

### Analysis of the relationships between MetS incidence and its components and anthropometric indices

Relationships between individual MetS components and anthropometric indices were assessed using regression analysis. Table 2 shows the model coefficients β and standard errors (SE).

**Table 2:** Linear regression models of the relationship between the anthropometric indices and MetS incidence.

Dependent variable	Independent variable	Simple model		Adjusted model	
	MetS component	β (95% CI)	Se	β (95% CI)	Se
BMI	HDL	− 0.233 (− 0.381 ÷ − 0.069)	0.075	− 0.206 (− 0.355 ÷ − 0.057)^a^	0.076
	Glucose	0.182 (0.073 ÷ 0.291)^b^	0.055	0.188 (0.081 ÷ 0.296)^a^	0.055
	Triglycerides	0.137 (− 0.014 ÷ 0.288)	0.077	0.099 (− 0.054 ÷ 0.251)	0.077
	HBP or AHT	0.264 (0.162 ÷ 0.366)^b^	0.052	0.232 (0.128 ÷ 0.335)^a^	0.053
	WC	0.843 (0.786 ÷ 0.900)^b^	0.029	0.843 (0.784 ÷ 0.901)^a^	0.030
BRI	HDL	− 0.231 (− 0.379 ÷ − 0.082)^b^	0.075	− 0.191 (− 0.338 ÷ − 0.043)^a^	0.075
	Glucose	0.263 (0.156 ÷ 0.370)^b^	0.054	0.270 (0.165 ÷ 0.375)^a^	0.053
	Triglycerides	0.198 (0.048 ÷ 0.348)^a^	0.076	0.153 (0.004 ÷ 0.303)^a^	0.076
	HBP or AHT	0.330 (0.230 ÷ 0.430)^b^	0.051	0.294 (0.193 ÷ 0.395)^a^	0.051
	WC	0.934 (0.896 ÷ 0.972)^b^	0.019	0.927 (0.888 ÷ 0.966)^a^	0.020
ABSI	HDL	− 0.056 (− 0.209 ÷ 0.096)	0.077	− 0.022 (− 0.177 ÷ − 0.132)	0.078
	Glucose	0.198 (0.089 ÷ 0.307)^b^	0.055	0.201 (0.092 ÷ 0.310)^a^	0.055
	Triglycerides	0.141 (− 0.010 ÷ 0.292)	0.077	0.118 (− 0.037 ÷ 0.273)	0.079
	HBP or AHT	0.191 (0.087 ÷ 0.295)^b^	0.053	0.172 (0.066–0.278)^a^	0.054
	WC	0.526 (0.436 ÷ 0.616)^b^	0.046	0.515 (0.421 ÷ 0.609)^a^	0.048
LAP	HDL	− 0.398 (− 0.540 ÷ − 0.255)^b^	0.072	− 0.351 (− 0.494 ÷ − 0.209)^a^	0.072
	Glucose	0.181 (0.027 ÷ 0.335)^a^	0.078	0.168 (− 0.021 ÷ 0.316)^a^	0.075
	Triglycerides	0.866 (0.789 ÷ 0.942)^b^	0.039	0.840 (0.764 ÷ 0.916)^a^	0.039
	HBP or AHT	0.147 (− 0.004 ÷ 0.299)	0.077	0.088 (− 0.064 ÷ 0.240)	0.077
	WC	0.601 (0.479 ÷ 0.723)^b^	0.062	0.565 (0.439 ÷ 0.691)^a^	0.064

^a^*p* < 0.05; ^b^*p* < 0.01.

The adjusted model was adjusted for under harmful working conditions (UHWC).

According to both the simple model and the adjusted model, BMI was shown to be associated with glucose levels, high blood pressure (HBP) or arterial hypertension (AHT) and WC. The BRI showed a significant relationship with all MetS components in both models. For the ABSI, we were unable to identify any significant relationships with HDL or TG. The LAP index displayed a significant relationship with all MetS components except HBP or AHT. In the adjusted model, these dependencies continued to exist.

The odds ratios, 95% confidence intervals, sensitivities (Se) and specificities (Sp) of the simple and adjusted models are given in Table 3.

**Table 3:** The odds ratios and 95% confidence intervals for the influence of anthropometric indices on the probability of MetS occurrence.

Independent variable	Simple model			Adjusted model		
	OR	Se	Sp	OR	Se	Sp
BMI	1.213 (1.136–1.295)	32.5	86.0	1.208 (1.129–1.292)	30.6	80.8
BRI	2.235 (1.796–2.781)	42.1	84.6	2.145 (1.706–2.696)	41.8	81.3
ABSI	1.174 (1.108–1.244)	32.5	87.8	1.145 (1.077–1.217)	34.0	83.9
LAP	1.052 (1.036–1.069)	68.8	84.8	1.050 (1.034–1.067)	73.4	80.1

Table 3 shows that for our sample, the probability of MetS increased with increasing anthropometric indices by unity. A unit increase in BRI was associated with a 2.235-fold increase in the probability of MetS [95% C: (1.796–2.781)] in the simple model and with a 2.178-fold increase in the likelihood of MetS [95% CI (1.730–2.743)] in the adjusted model. Since none of the confidence intervals included 1, the associations between the indices and MetS were statistically significant.

The models showed the best sensitivity for the LAP index (68.8% for the simple model and 75.0% for the adjusted model), indicating their high predictive capability for detecting positive cases (presence) of MetS. The models for the ABSI showed the best specificity (87.8% for the simple model and 84.7% for the adjusted model), which points to their high ability to predict negative (absence of) MetS outcomes.

### Determining the threshold values for anthropometric indices

In this section, we focus the description of our results on graphic representation, which in this case was more informative than the corresponding* p* values.

**Figure 1:**
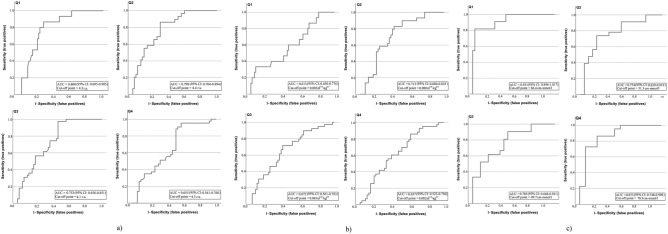
ROC analysis of anthropometric indices for age quartiles. (**a**) BRI; (**b**) ABSI; (**c**) LAP.

To compare the ability of the anthropometric indices to predict the presence of MetS, we performed an analysis of the ROC curves separately for each age quartile (Fig. 1).

Table 4 presents the characteristics of the models obtained from the ROC curve analysis.

**Table 4:** ROC curve analysis of different indices according to age quartile.

Anthropometric index	Q	AUC	Se	Sp	J	Overall model quality
BRI	1	0.800	0.867	0.290	0.577	0.690
	2	0.799	0.862	0.327	0.535	0.700
	3	0.753	0.974	0.481	0.494	0.600
	4	0.655	0.907	0.542	0.365	0.540
ABSI	1	0.613	0.333	0.058	0.275	0.450
	2	0.713	0.828	0.404	0.424	0.600
	3	0.672	0.718	0.385	0.333	0.550
	4	0.637	0.860	0.604	0.256	0.520
LAP	1	0.931	0.818	0.027	0.791	0.850
	2	0.774	0.739	0.200	0.539	0.630
	3	0.785	0.905	0.429	0.476	0.650
	4	0.873	0.727	0.071	0.656	0.750

Thus, based on the values obtained for the Youden index (J) and the area under the ROC curve (AUC), the BRI had the highest predictive value for men aged 41 to 52 years (Q_2_–Q_3_) (J = 0.494 and 0.535; AUC = 0.753 and 0.799). The best characteristics for the age quartiles Q_1_ and Q_4_ were found for LAP (J = 0.791 and 0.656; AUC = 0.931 and 0.873). The ABSI had the lowest J and AUC values and overall model quality; consequently, this index had the worst predictive ability in our study.

The threshold values of the anthropometric indices were determined by the Youden index. The cut-off points found by determining the maximum value of J for each age quartile served as a diagnostic threshold above or below which it was believed that the patient had or did not have MetS.

We used an approach based on heatmaps, a method for visualizing multidimensional data using intuitive graphical fills^34,35^.

**Figure 2:**
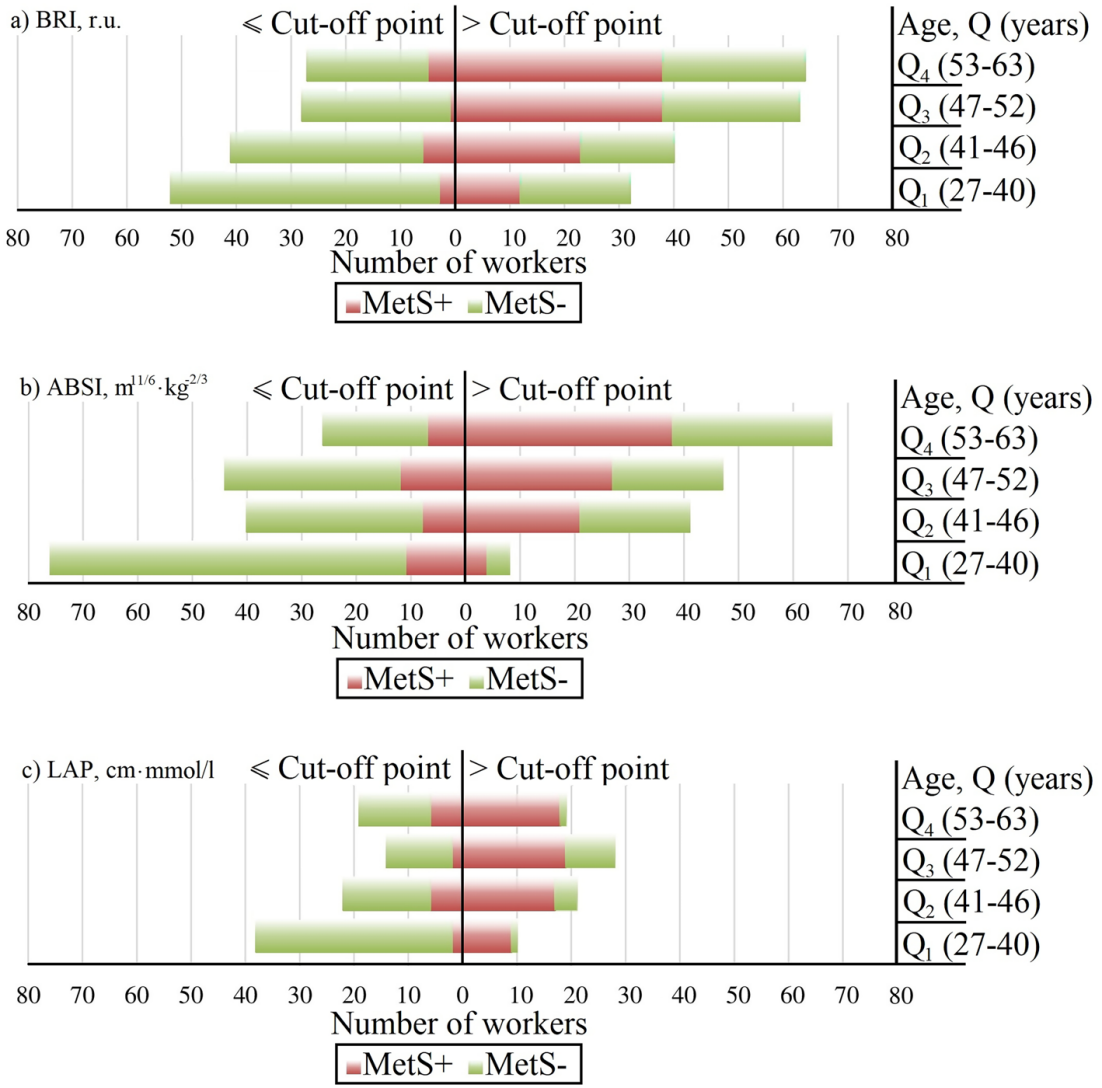
Distributions of worker numbers by age group and presence of MetS with a breakdown of the anthropometric indices by cut-off point. (**a**) BRI. (**b**) ABSI. (**c**) LAP.

Figure 2 shows the distributions of the number of workers by age group and the presence of MetS with a breakdown of the anthropometric indices by cut-off point.

The results should be interpreted by a diagram such as this: the vertical axis divides all workers into groups with index values below the threshold (left side) and above the threshold (right side). Shares of workers with MetS and without MetS are coloured in each age quartile (red and green, respectively). Therefore, we can estimate the quality of the "work of threshold" in each age quartile.

A vertical analysis of all three graphs shows that as the age quartile moves from the 1st quartile to the 4th quartile, the number of workers with MetS increases, which is expected since the incidence of MetS depends on age. The lowest number of MetS workers was in the first age quartile (27–40 years).

By analysing Fig. 2a–c horizontally, we can see that in the left part of the diagrams (≤ cut-off point), the number of metabolically healthy male workers (MetS-) is much greater than the number of people with metabolic syndrome (MetS +) in each age quartile.

In contrast, on the right side of the graphs, the number of MetS + employees exceeds the number of metabolically healthy employees. The only exception is the case of workers aged 27–40 years (Q_1_) with a BRI > the cut-off point, where the number of metabolically healthy men exceeds the number of MetS-positive cases.

In general, for all the anthropometric indices considered, the selected threshold values qualitatively determined the presence of MetS. It may be suggested that there is a relationship between elevated values of the anthropometric indices considered (> cut-off point) and the risk of MetS. Moreover, it is obvious that the quality of life for predicting the presence of MetS varies somewhat with age.

The implications of the results for clinical practice—the cut-offs proposed in this study provide an earlier diagnosis of MetS than the commonly accepted obesity criterion (i.e., BMI ≥ 30 kg/m^2^).

The implications of the results for occupational health:

- improving tactics for conducting routine preventive medical examinations and providing medical care to the working population in terms of early detection of occupational morbidity and occupational diseases (including cardiovascular diseases);
- implementation of personalized and individual risk-oriented medical and preventive programs for workers in harmful and/or dangerous working conditions.

## Discussion

The predictive capacity of several classical and new anthropometric indices for the detection of MetS in male workers employed in hazardous working conditions was first explored. The results of the study confirm the usefulness of these indicators for predicting the risk of developing MetS. A growing body of evidence now suggests that regional fat accumulation is much more important than general obesity in stratifying the risk of cardiometabolic disorders. According to data from the literature, a significant role of adipose tissue in obesity is played by morphological changes in visceral adipose tissue, as well as by the processes of remodelling and inflammation followed by the development of dysfunction^36^. Klisich together with his colleagues, in several of their studies, concluded that a simple anthropometric indicator of obesity, such as BMI, should not be decisive in the verification of obesity, and risk stratification based solely on this indicator is insufficient for a comprehensive assessment of the likelihood of developing diseases associated with obesity^37,38^.

Moreover, an increasing number of researchers believe that simple and inexpensive anthropometric measurements can and should be used to predict MetS. In addition to the classical BMI and WC, which have been used in clinical practice for decades, new indicators, such as the BRI, ABSI and LAP, have emerged. This does not mean abandoning the existing methods employed for accurately determining body composition, such as computed tomography, magnetic resonance imaging, bioelectrical impedance analysis, dual-energy X-ray absorptiometry, hydrostatic weighing and plethysmography^39–44^. However, despite their advantages in terms of accuracy, their use in clinical practice is limited by financial costs, time consumption, and the need for specially trained personnel. This issue is especially relevant for cases where it is necessary to assess the risk of MetS in the medical unit of an industrial enterprise.

In the present study involving 347 male workers employed in hazardous working conditions, the study revealed a relationship between anthropometric indices and the presence of both MetS and its individual components (see Fig. 2). The prevalence of MetS in our sample was 36.3%, which is somewhat higher than that reported in the WHO data for the adult population^2^. We believe that this higher incidence is due to employment under harmful working conditions, which has been shown to be related to the incidence of metabolic disorders in many epidemiological studies^27–29^.

Our study revealed a direct relationship between MetS and anthropometric indices such as BMI, BRI, ABSI, and LAP with and without adjustment for UHWC. For the sample under consideration, the probability of MetS increased with increasing anthropometric indices (see Table 2). Moreover, the results of previous studies on the relationship between anthropometric indices and MetS are contradictory. Thus, in adult Iranians, BMI was found to be a better predictor of MetS than ABSI^45^. In our study, based on the results of the regression analysis, the BRI performed better than BMI, ABSI, or LAP in predicting MetS incidence. Increasing BRI by unity was associated with a 2.235-fold increase in the probability of MetS; 95% CI (1.796–2.781). Our results support those of the MetS study among Peruvian adults^46^, in which the BRI performed similarly to or better than BMI in the prediction of MetS. A unit increase in the BRI in their study was associated with a 2.43-fold increase in the likelihood of MetS; 95% CI (1.95–3.02). In our study, BMI was the second most common factor in terms of predictive power, with an OR = 1.213 and a CI of 1.136–1.295, and the third most common factor was the ABSI, with an OR = 1.174 and a CI of 1.108–1.244, followed by LAP.

Regarding the relationship between individual components of MetS and anthropometric indices, in a large cross-sectional study carried out in the USA, Mooney et al. reported that BMI was the best predictor of BP, while central obesity measures (including WC) were the best predictors of fasting glucose^47^. Researchers in China reported that the BRI performed similarly to WC and BMI as predictors of diabetes mellitus, and all of these indices outperformed the ABSI^48^.

The results of our study are similar: the BRI was found to have a statistically significant positive relationship with all MetS components. A statistically significant positive relationship was detected between BMI and fasting glucose, HBP or AHT, and WC. For the ABSI, there was a statistically significant relationship with all MetS components, except for the level of triglycerides in the blood. The LAP index was found to have a statistically significant positive relationship with all MetS components except HBP and AHT. In the model adjusted for UHWC, these dependencies persisted, with only a slight decrease in the β coefficients.

We were unable to find any studies that determined the reference values of the BRI, ABSI, or LAP indices for Caucasian men employed in hazardous work conditions. We chose the cut-off points found using the Youden index of the indices as diagnostic thresholds above or below which the patient was considered to have MetS or not.

Models for predicting the presence of MetS in subjects built on the basis of the cut-off point of each index showed good sensitivity and specificity. The highest values occurred in the model built for LAP for the third age quartile: Se = 90.5%, Sp = 42.9%. The BRI for the same age quartile, Q_3_, had Se = 97.4% and Sp = 48.1% and was second in terms of model quality. The lowest sensitivity and specificity values were displayed by the ABSI-built model for the first age quartile: Se = 33.3%, Sp = 5.8%. Our study demonstrated the usefulness of anthropometric indices as markers of cardiometabolic health in male industrial workers employed in hazardous working conditions.

Our results are similar to those obtained by researchers in China, who determined threshold values for the BRI and ABSI indices for a sample of Chinese adults^8^. In their study, the cut-off points for men of all considered age categories were somewhat less than those in our sample (3.388–3.929 r.u. compared to 4.1–4.4 r.u.). A similar situation can be observed for the ABSI cut-off points: 0.077–0.080 m^11/6^ kg^−2/3^ in the Chinese study and 0.080–0.083 m^11/6^ kg^−2/3^ in our study.

Moreover, the ABSI threshold found in our study (0.080) was the same as the sex-specific cut-off points for ABSI among adult men in another study in China and in Poland^49,50^.

Regarding the cut-off points for the BRI, our results differ from the findings of the Polish study: 4.1 for the age quartile Q_3_ and 4.4 for the age quartile Q_2_ in our study, compared to 4.82 in the Polish study^50^.

A comparison of our LAP cut-off point for men working under hazardous working conditions with the results of a study of markers for predicting MetS in middle-aged and elderly Chinese workers revealed significant differences: 37.99 cm mmol/l in China and > 49.7 cm mmol/l in our study^20^.

Despite the heterogeneity of the population characteristics and geographic differences, the results of our study and other studies show that simple anthropometric measurements are of global value in identifying individuals at high risk of developing metabolic syndrome.

One of the strengths of this study is the homogeneity of the sample in terms of social status, income, educational level, and gender. The participants in the study were male industrial workers employed in hazardous working conditions (common to most subjects).

This circumstance allowed us to study the relationships between the indices and MetS components in pure form (not distorted by covariates). Another strength is the size of the sample, which was sufficient to draw valid conclusions. All anthropometric and BP measurements and laboratory investigations were performed by trained medical personnel according to standardized protocols, allowing us to exclude the possibility of bias.

In interpreting the findings of this study, several limitations should be taken into account. This was a cross-sectional study by design, so the results cannot reflect causal relationships. Serum triglycerides and HDL were measured in a subgroup of 169 workers. Therefore, the LAP index was calculated for 169 workers.

No statistically significant differences were detected in the frequency of smoking between the participants. Introducing the variable "smoking" into the regression models as a covariate did not provide a significant result. For this reason, smoking status was not adjusted for these patients.

In summary, the results of the present study, carried out on a representative sample, confirm and contribute to the body of evidence supporting the usefulness of the new anthropometric indices for predicting the risk of MetS in male workers in industrial enterprises in hazardous work environments.

## Conclusions

The relationships between several classic and new anthropometric indices and MetS have been analyzed. The presence of MetS and its components has a statistically significant effect on the variation in the new anthropometric indices considered. The latter, therefore, can serve as effective indicators of cardiometabolic risk factors for industrial workers employed in hazardous working conditions.

The results of this study suggest that it is advisable to use new anthropometric indices, which have good predictive capacity and are simple and easy to apply. The cut-off values obtained in this study may be very useful for screening and prevention of MetS in industrial environments. Finally this will allow to reduce the level of occupational diseases, the level of disability, and the number of sudden deaths at work due to cardiometabolic problems. All of the above are cost-effective for society—savings in health care costs and higher labor productivity can offset and exceed the direct investment required to implement these activities.

Further studies should involve other categories of subjects, in particular working women, as well as non-working, retired population, to allow patient stratification by level of cardiometabolic risk and subsequent development of a range of preventive measures to improve diagnostic practice and treatment strategies. Also, future studies are needed to determine cut-offs based on ethnicity.

## Data Availability

Data are available from the authors upon reasonable request.
